# Association of the functional ovarian reserve with serum metabolomic profiling by nuclear magnetic resonance spectroscopy: a cross-sectional study of ~ 400 women

**DOI:** 10.1186/s12916-020-01700-z

**Published:** 2020-08-31

**Authors:** Karema Al Rashid, Amy Taylor, Mary Ann Lumsden, Neil Goulding, Deborah A. Lawlor, Scott M. Nelson

**Affiliations:** 1 School of Medicine, Glasgow Royal Infirmary, New Lister Building, University of Glasgow, Glasgow, G31 2ER UK; 2 MRC Integrative Epidemiology Unit at the University of Bristol, Bristol, BS8 2BN UK; 3 Population Health Science, Bristol Medical School, Bristol, UK; 4 NIHR Bristol Biomedical Research Centre, Bristol, UK

**Keywords:** Ovarian reserve, AMH, AFC, Metabolomics

## Abstract

**Background:**

Women with diminished ovarian reserve are known to have increased cardiovascular risk, whether there is a continuous association between the ovarian reserve biomarkers; anti-Müllerian hormone (AMH), antral follicle count (AFC) and cardio-metabolic risk factors are unknown.

**Methods:**

A cross-sectional study of 398 women intending to undergo IVF with pre-treatment early follicular AMH and AFC measurements. Serum lipids, lipoprotein subclasses and low-molecular-weight metabolites were quantified by NMR spectroscopy (155 metabolic measures). Associations were analysed using multivariable regression.

**Results:**

Participants were mean 35.5 (SD 4.43) years old and had a median AMH of 16 pmol/l (IQR 8.8, 28.0 pmol/l) and a median AFC of 12 (IQR 7.16). AMH showed positive associations with HDL, omega-6 and polyunsaturated fatty acids and the amino acids isoleucine, leucine and tyrosine, with effects ranging from 0.11 (95%CI 0.004 to 0.21) for total lipids in small HDL to 0.16 (0.06 to 0.26) for isoleucine, for a mean difference of one SD of metabolite per one SD increment in AMH, and negatively with acetate: − 0.31(− 0.22, − 0.004) SD per 1 SD AMH. AFC was positively associated with alanine, glutamine and glycine. Results were consistent, though less precisely estimated, when restricted to those women who were preparing for treatment because of their partner’s infertility.

**Conclusions:**

In women intending to have IVF, AMH and AFC were not associated with traditional lipid measured but were associated with a number of novel cardiovascular risk factors. Prospective studies will be required for replication, determination of causality and confirmation that ovarian reserve is impacting on metabolism rather than variation in metabolism is influencing ovarian reserve.

## Background

Female reproductive ageing is the result of a gradual decrease in both the quantity and quality of oocytes [1]. Genetic, environmental and lifestyle factors are all known to contribute to the timing and depletion of the ovarian reserve. Markers of diminished ovarian reserve, such as low anti-Müllerian hormone (AMH) and low antral follicle count (AFC), have been shown to associate with earlier menopause [2, 3]. Several observational studies have investigated the association between these markers of diminished ovarian reserve and cardiovascular risk factors, with several [4–8] but not all studies [9–11], suggesting that a diminished ovarian reserve may be associated with an unfavourable circulating cardiometabolic risk profile and cardiovascular events. However, these studies have been limited to a restricted number of established cardiovascular risk factors, including evaluation of total and LDL cholesterol [6], homeostatic model assessment as a surrogate for insulin resistance (HOMA-IR) [12] or have considered these established risk factors together as a composite outcome of cardiometabolic risk [4, 5].

Detailed metabolic profiling or metabolomics has been applied successfully to identify novel biomarkers for the development of cardiovascular disease [13, 14] and all-cause mortality [15], with improved prediction as compared to models containing conventional risk factors [15]. Serum nuclear magnetic resonance (NMR) metabolomics which enables reproducible quantification of circulating lipids and abundant metabolites [16] has facilitated its use in the assessment of the changes in metabolites with adiposity [17], glycaemia [18], pregnancy [19] and menopausal status [20].

The aim of the current study was to assess the association of ovarian reserve (as measured by AMH and AFC) with 155 circulating metabolic measures. These measures were profiled by a high-throughput NMR metabolomics platform, covering a wide range of metabolic pathways including lipoprotein lipids, fatty acids, amino acids, ketone bodies and glycaemic traits, which are highly relevant to cardiometabolic risk and overall health.

## Methods

### Study design and participants

This is a cross-sectional study of women aged 18 to 45 who presented at Glasgow Royal Infirmary, UK, for assessment prior to assisted conception between 1 April 2017 and 31 March 2019. Exclusion criteria were a documented positive pregnancy test at the time of presentation, body mass index (BMI) ≥ 35 kg/m^2^ and/or requiring oocyte or embryo donation. A total of 400 women were recruited, and of these, 398 (99%) had complete data on AMH, AFC and at least one NMR metabolite and were included in the analyses presented in this paper.

The study was conducted according to the ICH Guideline for good clinical practice, the Declaration of Helsinki and the Convention of the Council of Europe. All women provided written informed consent. The study protocol was approved prior to study initiation by the relevant institutional review boards (see Supplementary Material).

### Study procedures

Demographic, lifestyle, fertility and medical history was obtained by self-reported questionnaire and clinical data by linkage to electronic medical records.

AFC was determined by two- or three-dimensional transvaginal ultrasound (AFC was defined as the total number of antral follicles with a size of 2–10 mm in both ovaries) on menstrual cycle days 2–4. Follicle counts greater than 20 follicles per ovary were classed as ≥ 20, consistent with the diagnostic threshold for PCOS, and that ovary was not counted further [21]. Due to the potential for inter-sonographer variability, sonographers were provided with training and were asked to follow published practical recommendations for accurate transvaginal ultrasound [22]; each sonographer used standard equipment (Acuson Sequoia, Siemens Germany).

Non-fasted blood samples were collected during the same visit that transvaginal ultrasound was performed. AMH was measured using the Beckman Coulter AMH automated method on a clinically validated immunoassay platform (Access 2, Beckman Coulter, USA). The assay was calibrated and quality controlled using the manufacturer’s reagents and is known to have a measuring range of 0.08–24 ng/ml (0.57–171 pmol/l). The limit of quantitation (LoQ) was 0.02 ng/ml (0.014pmo/l), with the 20% CV LoQ 0.08 ng/ml (0.57 pmol/l). The coefficient of variation between runs for two levels of control ran at < 4.4%.

Additional blood samples were taken for NMR analyses and immediately spun and frozen at − 80 °C, and all NMR assays completed for this study were undertaken within 1 year of storage and with no previous freeze/thaw cycles.

### NMR protocol

Profiling of 155 lipid and metabolite measures was performed by a high-throughput targeted NMR platform [Nightingale Health© (Helsinki, Finland)] at the University of Bristol. The platform applies a single experimental setup, which allows for the simultaneous quantification of routine lipids, 14 lipoprotein subclasses and individual lipids transported by these particles, multiple fatty acids, glucose, the glycolysis precursors lactate and pyruvate, ketone bodies and amino acids in absolute concentration units (mostly mmol/l). The NMR-based metabolite quantification is achieved through measurements of three molecular windows from each sample. Two of the spectra (LIPO and LMWM windows) are acquired from native serum and one spectrum from serum lipid extracts (LIPID window). The NMR spectra were measured using Bruker AVANCE III spectrometer operating at 600 MHz. Measurements of native serum samples and serum lipid extracts are conducted at 37 °C and 22 °C, respectively. Details of this platform have been published previously [16, 23], and it has been widely applied in genetic and observational epidemiological studies [13–15, 17, 19, 20, 24–26]. Further details of the platform are provided in Additional file 1 (Supplemental text, Table S1 and Fig. S1 [16]).

### Metabolite quantification and quality control

The NMR spectra were analysed for absolute metabolite quantification (molar concentration) in an automated fashion. For each metabolite, a ridge regression model was applied for quantification in order to overcome the problems of heavily overlapping spectral data. In the case of the lipoprotein lipid data, quantification models were calibrated using high-performance liquid chromatography methods and individually cross-validated against NMR-independent lipid data. Low-molecular-weight metabolites, as well as lipid extract measures, were quantified as millimoles per litre based on regression modelling calibrated against a set of manually fitted metabolite measures. The calibration data are quantified based on iterative line-shape fitting analysis using the PERCH NMR software (PERCH Solutions Ltd., Kuopio, Finland). Absolute quantification cannot be directly established for the lipid extract measures due to experimental variation in the lipid extraction protocol. Therefore, serum extract metabolites are scaled via the total cholesterol as quantified from the native serum LIPO spectrum.

### Assessment of potential confounders

**Fig. 1:**
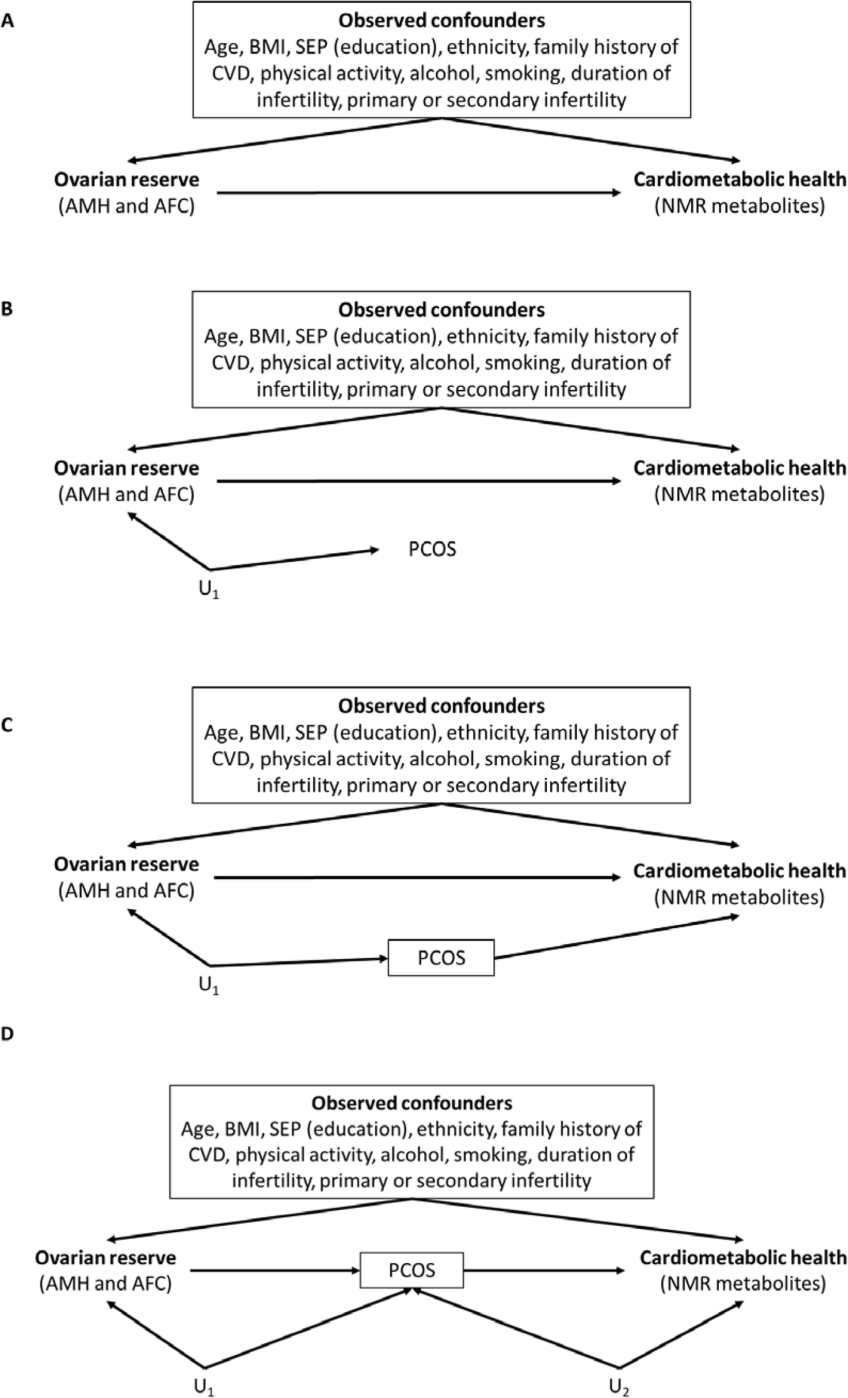
Directed acyclic graphs (DAGs) of the association of ovarian reserve with cardiometabolic health. **a** Our main analyses, in which we a priori considered factors that were known to, or highly plausibly influenced ovarian reserve and cardiometabolic health, as confounders to be controlled for. **b**–**d** Further consider whether we should or should not control for PCOS. In all of these, we assume that there are underlying unmeasured factors that generate an association between ovarian reserve and PCOS (U_1_). For example, it has been suggested that common genetic and/or intrauterine factors affect both of these. **b** We assume that PCOS is not causally related to cardiometabolic health (no arrow from PCOS to cardiometabolic health). **c** PCOS does causally influence cardiometabolic health and that it is on a confounding path between ovarian reserve and cardiometabolic health via U_1_. **d** Assume that PCOS causally influences cardiometabolic health but that it is a mediator between ovarian reserve and cardiometabolic health. Deciding whether we need to adjust for PCOS therefore depends on evidence for a causal link between PCOS and cardiometabolic health and if there is a link evidence as to whether PCOS is likely to be a confounder or a mediator. In relation to point 1, whilst there have been several observational studies showing an association of PCOS with cardiometabolic health, recent Mendelian randomisation studies suggest that PCOS does not causally influence type 2 diabetes, coronary heart disease or stroke (making **b** a plausible scenario) [31]. If the scenario depicted in **b** is correct, then controlling for PCOS is not necessary, but if done, it should have no impact on the results. It is not uncommon in observational epidemiology to have a risk factor for an outcome and be unsure which is more plausible—that it is a confounder or a mediator. If PCOS is on the confounding path between unmeasured factors (U_1_) as shown in **c**, we would definitely want to adjust for it as this would be the only way to block this confounding (given U_1_ variables are unmeasured). However, if PCOS is a mediator (**d**), then we would not want to adjust for it. Primarily, this is because we want to know the ‘total’ potential effect of ovarian reserve on cardiometabolic health and not remove any of that going via mediation. It is also possible that adjusting for a mediator can introduce what is known as collider bias [32]. If there are unmeasured confounders of the mediator (PCOS) and cardiometabolic health (shown by U_2_), then adjusting for PCOS could generate spurious associations between ovarian reserve and cardiometabolic health

In relation to our analyses and control for confounders, we used currently recommended practice of defining confounders a priori (before undertaking analyses) using directed acyclic graphs (DAGs) [27, 28]. This approach defines confounders as any characteristic that is known to cause variation in the exposure (here ovarian biomarkers AMH and AFC) and outcome (NMR metabolites) or is plausibly a cause of exposure and outcome. Using a priori knowledge and relevant literature, our selected confounders were age, BMI, educational attainment, ethnicity, family history of cardiovascular disease (defined as first-degree relative affected) physical activity, alcohol intake, smoking status, duration and cause of infertility and whether infertility was primary or secondary (with secondary defined as a woman unable to establish a clinical pregnancy but who has previously been diagnosed with a clinical pregnancy) [29]. These are known to or plausibly influence both ovarian reserve and the NMR metabolites [30]. Figure 1a shows the DAG for this, our main, confounder-adjusted analyses. Following reviewer comments, we also considered the extent to which PCOS might be part of a confounding path and whether we should also adjust for it. There is evidence ovarian reserve and PCOS may share underlying common causes, including genetic variation and intrauterine exposures. If these, specific genetic variants and exposures are also related to cardiometabolic health, then PCOS may be on a confounding path between ovarian reserve and cardiometabolic health. Three possibilities are considered in Fig. 1b–d. Whether or not to adjust for PCOS is in part related to the fact that we do not have genetic or intrauterine data on the women included in this study, and so, these would be potential unmeasured confounders. In 1B, we assume that there is no causal relationship between PCOS and cardiometabolic health. Whilst observational studies have shown associations of PCOS with type 2 diabetes, and some also show associations with CHD, a recent Mendelian randomisation study has suggested no causal effect of PCOS on type 2 diabetes, coronary heart disease or stroke [32], making this scenario plausible. In this scenario, PCOS is not on a confounding path, and adjusting for it should not alter the results. Figure 1c and d suggest PCOS does causally influence cardiometabolic health. In 1C, PCOS is on a confounding path, and we would want to adjust for it. In 1D, it is on a mediating path, and we would not want to adjust for it. Given we cannot be certain which of these scenarios is correct in additional analyses we, repeat all of our main analyses with those with known PCOS removed. We chose to remove them rather than add to a multivariable model because there were only 24 cases. Weight and height [used to calculate the body mass index (BMI)] were measured in light clothing and unshod. Weight was measured to the nearest 0.1 kg using Tanita scales; height was measured to the nearest 0.1 cm using a Harpenden stadiometer. Smoking status was categorised as ever versus never (to be considered for state-funded assisted conception, women had to have not smoked for at least 3 months, and this was confirmed by a negative cotinine breath test). All other confounders, including an existing diagnosis of PCOS, were obtained by a questionnaire or from medical notes when the women were originally recruited. Full details of the assessment of these confounders are provided in the supplementary material.

### Statistical analysis

All analyses were conducted using R version 3.4.2 (R Foundation for Statistical Computing, Vienna, Austria). An analysis plan was written in June 2019. Characteristics were summarised as *n*, total range, mean, standard deviation, median and 25th and 75th quantiles (IQR) as appropriate. Multivariable linear regression was used to examine the associations of functional ovarian reserve markers (treated as exposures) with serum metabolic profiles (treated as outcomes). Robust standard errors were estimated for all associations, as some metabolite concentrations had skewed distributions. The metabolic measures were scaled to standard deviation (SD) units (by subtracting the mean and dividing by the standard deviation of all women included in the analyses). This scaling allows easy comparison of multiple metabolic measures with different units or with large differences in their concentration distributions. AMH and AFC were also scaled to SD units in the same way as the metabolites. This was done for ease of comparison of results between the two biomarkers. Associations were adjusted for all a priori selected confounders (age, ethnicity, education, family history of cardiovascular disease, BMI, physical activity, alcohol, smoking, duration of infertility and whether the woman had primary or secondary infertility).

#### Additional analyses

In addition to presenting our main results as the difference in mean metabolite in SD units per SD of AMH or AFC, we also present the full results (confounder adjusted) in the metabolite, AMH and AFC original units in the supplementary material. One woman was taking lipid-lowering medication, and removal of her from the analyses did not alter any of the findings from the main results (results available from the corresponding author on request). We repeated our main analyses only on those women with a known partner cause of infertility (*N* = 87 (22% of the cohort). We knew a priori that this sample size would lack statistical power for reliable results but wanted to compare the point estimates in this group to the results of the whole cohort to provide some indication as to whether our results might be driven by the cause of infertility in the women or potentially generalisable to women of reproductive age without infertility. We compared the magnitudes of the results in women who were going to undergo assisted conception because of partner infertility with those of the whole cohort using a scatterplot. As 99% of the women recruited had full data on ovarian reserve and at least one metabolite, with < 1% of these having missing covariable data (see Table 1), we did not need to undertake any additional analyses to explore potential biases due to missing data. To explore the departure from linearity where there was evidence of an association, AMH and AFC were split into quarters of their distribution and regression models were run with these quarters as a continuous score and as a 4 level categorical variable (with 3 indicators). A likelihood ratio test was used to compare these two models. Statistical support that the second (3 indicators) model was a better fit of the data would suggest a possible non-linear association.

**Table 1:** Baseline characteristics of the study population (*N* = 398)

**Age (years), mean, SD, range**	35.5 (4.43) 22–45
**Ethnicity,*N*(%)**	
White European	365 (92%)
Asian	28 (7%)
Others	5 (1%)
**Highest education,*N*(%)**	
School leaving exams	185 (46%)
Undergraduate degree	139 (35%)
Postgraduate degree	74 (19%)
**BMI, mean, SD, range**	24.7 (3.2) 18.2–32.5
**Ever smoked,*N*(%)**	104 (26%)
**Alcohol (units per week), median, IQR, range**	4 (1, 8) 0–27
**Physical activity (times per week),*N*(%)**	
Never	12 (3%)
Once	29 (7%)
Twice	86 (22%)
3–4 times	239 (60%)
5–7 times	25 (6%)
7+ times	7 (2%)
**Family history of cardiometabolic disease,*N*(%)**	208 (52%)
**Gravidity, median, IQR, range**	0 (0, 1) 0–12
**Parity, median, IQR, range**	0 (0, 0) 0–4
**Duration infertility (years), median, IQR, range**	3 (2, 4) 1–13
**Primary infertility,*N*(%)**	271 (68%)
**Cause of infertility**	
Unexplained	203 (51%)
Tubal disorder	44 (11%)
Endometriosis	32 (8%)
Ovulatory disorder	24 (6%)
Male factor/no male partner	87 (22%)
Others	8 (2%)
**First IVF cycle (missing data,*N*** **= 3)**	376 (95%)
**AMH, median, IQR, range**	16.1 (8.9, 28.0) 1–170.8
**Total AFC, median, IQR, range**	12 (7, 16) 0–40

In cases where endometriosis and tubal disorders were both given as the cause of infertility (*N* < 5), half were randomly assigned to tubal disorders and half to endometriosis

#### Accounting for multiple testing

Due to the correlated nature of the metabolic biomarkers, over 95% of the variation in the 155 metabolic biomarkers was explained by 14 principal components. Therefore, multiple testing correction, accounting for 14 independent tests using the Bonferroni method, resulted in *p* < 0.0036 (0.05/14) being denoted as statistically significant.

## Results

Three hundred and ninety-eight women (99% of the 400 recruited) with available AMH and AFC levels and data on at least one metabolite were included in the study. The characteristics of the participants are shown in Table 1. Mean (SD) age of the women was 35.5 (4.4) years, and the majority (92%) were White European, with over 50% having a university degree, 26% being ever smokers, 68% exercising more than 3–4 times per week, 52% having a family history of cardiovascular disease and median alcohol consumption being 4 units per week. Mean (SD) BMI was 25 (3) kg/m^2^, and 95% were due to start their first treatment cycle, with 49% having an unknown cause of infertility and 24% a cause related to their partner. The median AMH was 16.1 pmol/l (IQR 8.8, 28.0 pmol/l) and median AFC of 12 (IQR 7, 16). AMH and AFC were positively correlated (Spearman’s correlation coefficient = 0.55, *p* < 0.001).

The unadjusted associations of AMH and AFC with selected confounders are shown in Additional file 1 Tables S2 and S3, respectively. Age was negatively associated with AMH (difference in mean − 0.09 SD per 1-year older age, 95% CI − 0.11, − 0.07) and AFC (− 0.06 SD per 1-year older age, 95% CI − 0.08, − 0.04). An ovulatory cause of infertility compared with an unexplained cause was associated with a higher AMH (difference in mean 0.93 SD, 95% CI 0.48, 1.37) and AFC (0.65, 95% CI 0.20, 1.11). Secondary infertility was strongly negatively associated with AFC (− 0.34SD (95% CI − 0.55, − 0.13), with a weaker negative association with AMH (− 0.11 (95%CI − 0.32, 0.11)). Family history of cardiometabolic diseases was negatively associated, and non-White European ethnicity positively associated with AMH and AFC. There was no strong statistical evidence of an association between BMI or other potential confounders and either measure of ovarian reserve.

**Fig. 2:**
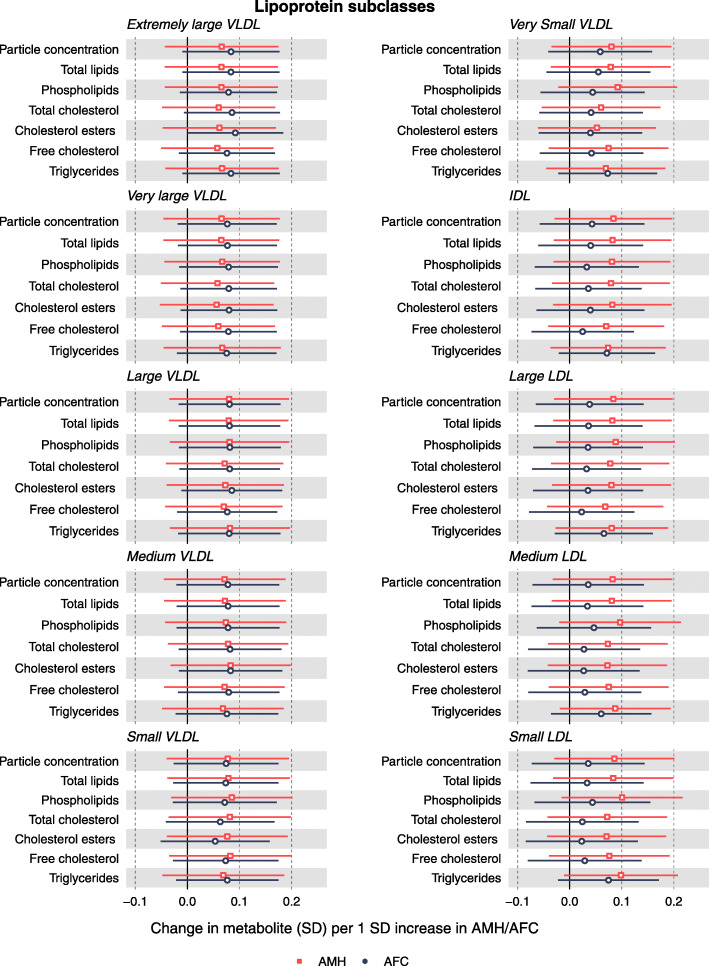
Associations of lipoprotein classes with AMH and AFC in women awaiting IVF. Effect sizes per 1 SD in metabolite concentrations and respective 95% confidence intervals are shown for AMH (red) and AFC (black). Adjusted for age, education, family history of CVD, BMI, physical activity, alcohol (units per week), ever smoking, ethnicity, duration of infertility, cause of infertility and primary/secondary infertility

**Fig. 3:**
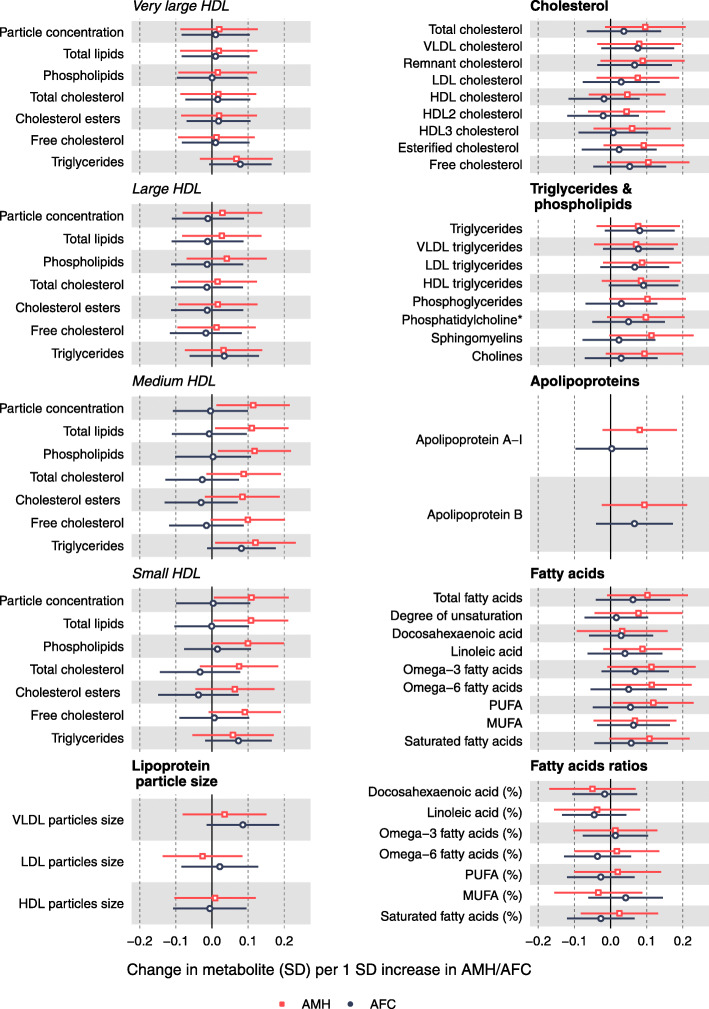
Associations of lipoprotein classes and fatty acids with AMH and AFC in women awaiting IVF. Effect sizes per 1 SD in metabolite concentrations and respective 95% confidence intervals are shown for AMH (red) and AFC (black). Adjusted for age, education, family history of CVD, BMI, physical activity, alcohol (units per week), ever smoking, ethnicity, duration of infertility, cause of infertility and primary/secondary infertility

**Fig. 4:**
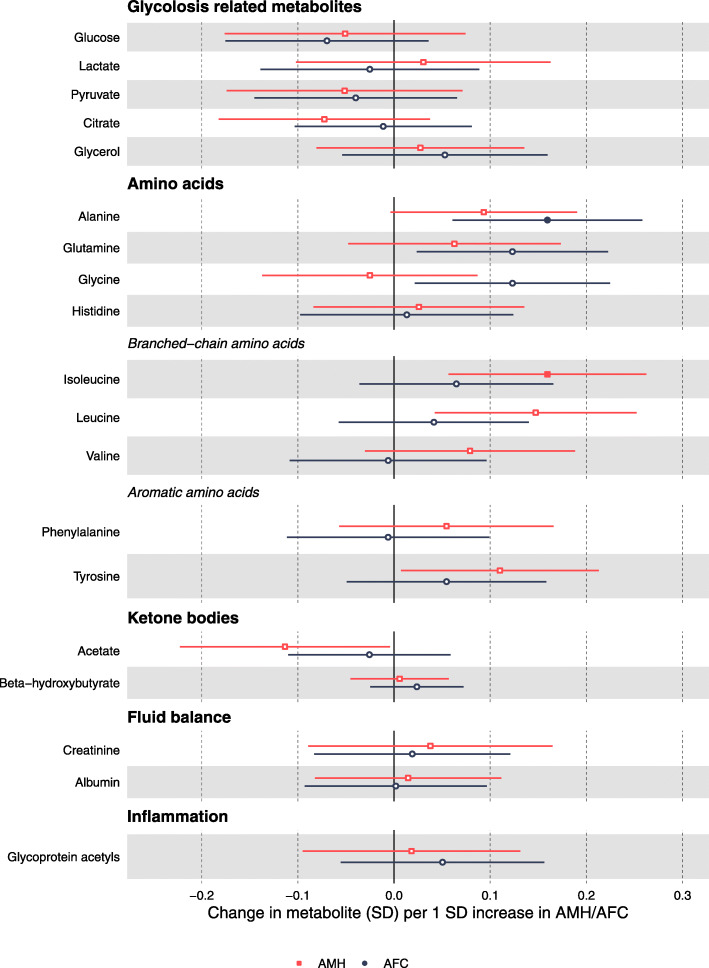
Associations of metabolic traits with AMH and AFC in women awaiting IVF. Effect sizes per 1 SD in metabolite concentrations and respective 95% confidence intervals are shown for AMH (red) and AFC (black). Adjusted for age, education, family history of CVD, BMI, physical activity, alcohol (units per week), ever smoking, ethnicity, duration of infertility, cause of infertility and primary/secondary infertility

The adjusted (for all potential confounders) associations between AMH and AFC and the respective metabolomics measures are SD units per SD of AMH/AFC and are presented in Figs. 2, 3 and 4. With the unadjusted associations presented Additional file 1 Figures S2 - S4. Confounder-adjusted results in the original units of AMH/AFC and the metabolites are shown in Additional file 2 Tables S4 and S5.

In confounder-adjusted analyses, AMH levels were positively correlated with measures of components of medium HDL and small HDL and with concentrations of omega-6 fatty acids and polyunsaturated fatty acids (PUFA). AMH was also positively associated with the amino acids isoleucine, leucine and tyrosine, and it was negatively associated with acetate concentrations. Although there were positive associations with lipoprotein subclasses, other cholesterol subtypes, glycerides and apolipoproteins, several of which had point estimates of ~ 0.1 SD or larger differences; these had wide confidence intervals that included the null and did not reach our multiple testing threshold for statistical significance.

Overall, the associations for AFC with the metabolic measures were in the same direction as those observed for AMH; however, they were weaker in magnitude and for many of them close to unity (Figs. 2, 3 and 4). The notable exceptions were the positive associations with the amino acids alanine, glutamine and glycine.

**Fig. 5:**
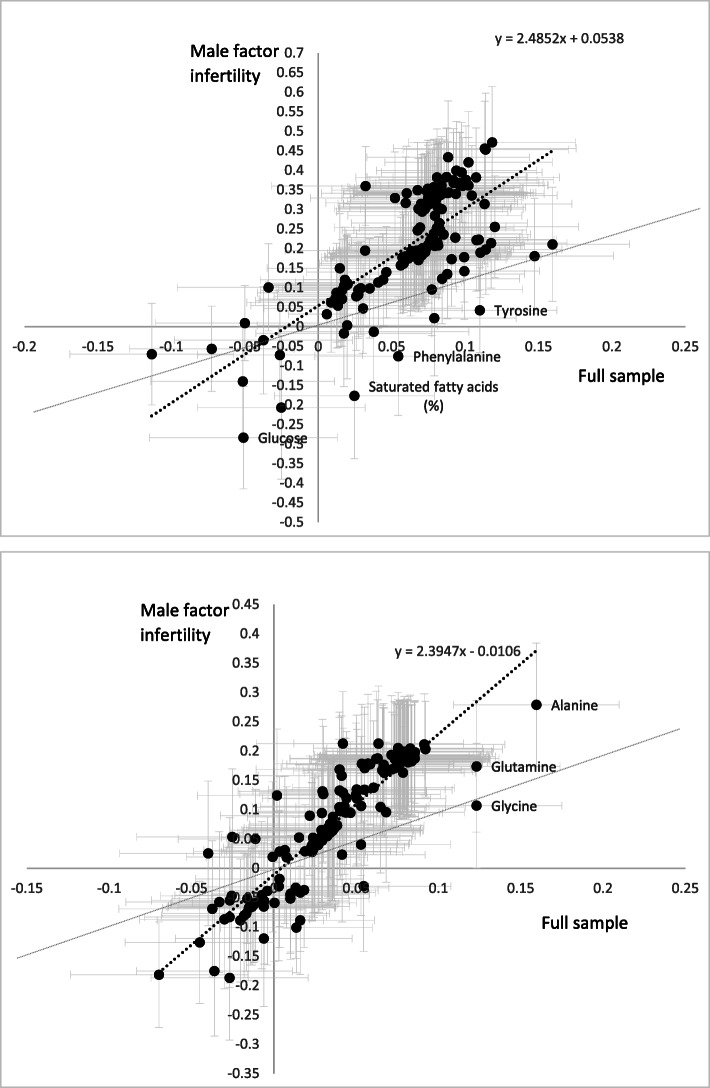
Scatterplot of the associations between AMH and AFC and metabolites in the full sample (*N* = 398) and subsample restricted to females with a reported male partner cause of infertility (*N* = 87). Figure 4a shows the associations with AMH; Fig. 4b shows associations with AFC

There was no strong evidence of departure from linearity for any of the observed associations (all likelihood ratio *p* values comparing a model with four categories as 3 indicator variables to the simpler model as a 4 level score ≥ 0.05). When analyses were repeated only in those undergoing assisted conception due to partner infertility (*n* = 87), most of the 155 associations of AMH were stronger than those in the main analyses including all women (Fig. 5a). This included the strengthening of the previously noted associations of AMH with the metabolomic markers. Positive associations were also observed for the various lipid concentrations within the medium and small very-low-density lipoproteins, intermediate-density lipoprotein and low-density lipoproteins, cholesterol, the mono-unsaturated fatty acids and alanine, with a negative association with glucose emerging; the negative association with acetate attenuated (Additional file 1 Figures S5-S7). In this subgroup, some associations of AFC also strengthened (Fig. 5b), with positive associations with various lipid concentrations across the size range of the very-low-density lipoproteins, triglycerides, apolipoprotein-B, mono-unsaturated fatty acids and the amino acids alanine and glutamine (Additional file 1 Figures S8-S10). When analyses were compared between the whole cohort and after exclusion of women with PCOS (*n* = 24), the associations were similar for AMH and AFC with correlations of 0.97 and 0.98, respectively (Additional file 1 Figure S11).

## Discussion

In this cross-sectional study of women attending a fertility clinic for evaluation, we identify several novel associations between the biomarkers of ovarian reserve, as measured by AMH and AFC, and circulating metabolites. Specifically, AMH showed positive associations with the amino acids isoleucine, leucine and tyrosine; medium HDL and small HDL; omega-6 fatty acids; and polyunsaturated fatty acids and a negative association with acetate. AFC had directionally consistent associations to those seen for AMH, but the magnitudes of association (measured on the same scale as SD difference in metabolite for a 1 SD increase in AMH or AFC) were weaker overall. Statistical evidence for positive associations with alanine, glutamine and glycine were observed. In our main analyses, including all women, we did not find strong statistical support for either biomarker being associated with an extensive lipid profile. This was still the case after the exclusion of women with PCOS. When we restricted analyses to the subgroup (22%) of women with male partner infertility, observed associations strengthened and we observed positive associations of AMH and AFC with a range of lipids, suggesting that the women infertility phenotypes may mask associations with lipid profiles.

Previous studies have suggested an association of lower ovarian reserve with vascular health [7, 33, 34]. Whether alteration in serum lipids contributes to this association is unclear. One small study (*n* = 50) reported a positive correlation between AMH and total cholesterol and LDL-C, and inverse correlation with HDL-C [35], whilst a larger study (*N* = 252) of women with PCOS found a weak positive correlation with HDL-C, which was attenuated when adjusted for BMI [36]. In contrast, larger studies of Chinese women did not observe an association between AMH and any of total cholesterol, LDL-C, HDL-C or triglycerides in either women with PCOS (*N* = 304), infertile women (*N* = 1896) [37] or in a general cohort of women (*N* = 6763) [38]. This is consistent with our own findings, with limited statistical evidence of an association between AMH and lipoprotein subclasses, lipoprotein particle size, cholesterol, LDL or HDL subtypes or triglycerides, phosphoglycerides, or apolipoproteins when the whole population was considered. Collectively, the evidence to date (including from our study) would support recent observational studies suggesting that the association of AMH with atherosclerosis may be independent of lipid levels [7, 33, 34]. As an alternative mechanism, it has been proposed that AMH has direct effects on cardiovascular tissue [39], making it more prone to injury and atherosclerosis [7]. Though specific evidence for this is lacking. We did not demonstrate associations between AFC and lipids, which have to our knowledge not been previously explored, further supporting suggestions that alternative mechanisms to dyslipidaemia may underlie the relationships between ovarian reserve and cardiovascular disease. Though as acknowledged below, the greater variability in AFC compared with AMH may have attenuated some of these results towards the null.

We report a positive association between AMH and higher circulating levels of PUFA and omega-6 fatty acids. However, a systematic review and meta-analysis found no evidence of either observational associations between dietary intake or measured circulating concentrations of omega-6 on cardiovascular diseases or any effect of dietary supplementation with omega-6 in randomised controlled trials [40], suggesting that even if the association of AMH with omega-6 is causal, this is unlikely to be a mechanism for preventing cardiovascular diseases.

Branch chain amino acids (BCAA), such as isoleucine, leucine and valine, have been found to be positively associated with a number of cardiometabolic risk factors, including adiposity, fasting glucose, insulin resistance, blood pressure, dyslipidaemia and indicators of coronary artery disease, in cross-sectional studies [41]. There are also positive associations of BCAA and aromatic amino acids with incident cardiovascular events in several large prospective studies [14, 42–44]. However, whether these are causally related is at present unknown. Our observed AMH-BCAA association is of interest, but the replication of our findings and confirmation of biological plausibility of causality would be required to assess whether the association of AMH with CVD may be mediated to some extent by BCAA.

The same NMR platform has previously been used to identify 14 metabolites associated with all-cause mortality in a meta-analysis of 12 cohorts and 44,168 participants [15], with subgroup analyses of 7603 participants identifying seven metabolites (XXL-VLDL-L, PUFA, lactate, histidine, leucine, phenylalanine and albumin) inversely associated with cardiovascular mortality and three (glucose, lactate and glycoprotein acetyls) positively associated with cardiovascular mortality [15]. Overall, our observed direction of associations was consistent with markers of low ovarian reserve relating to these metabolites that have previously been shown to robustly associate with cardiovascular disease mortality. Associations of AMH with PUFA and leucine met our adjusted statistical threshold. These findings, if replicated in larger cohorts, would support the overall concept that a diminished ovarian reserve may be associated with an unfavourable circulating cardiometabolic risk profile [5].

Our study has several strengths. To our knowledge, we are not aware of any study with a similar or larger sample size with detailed phenotypic and metabolite measurements. All women attended during the early follicular phase for the measurement of AMH and AFC. We included all women across the range of ovarian reserve including some women with extremely low and high AMH and AFC. We wrote, and have used, a prespecified analysis plan which incorporated adjustment for a wide range of a priori specified potential confounders. Subgroup analyses in those women who were awaiting assisted conception because of male partner infertility were also assessed, as these women may reflect a general population of women of reproductive age.

We do however acknowledge several limitations. Our analyses are cross-sectional and therefore could be explained by variation in metabolism (e.g. of amino acids) influencing ovarian reserve rather than the other way around, as we have assumed. Furthermore, we cannot assume that the small number of associations identified is causal. Residual confounding may have resulted from crude questionnaire measurements of physical activity, alcohol intake and family history of cardiovascular disease and the lack of any data on dietary intake. However, we adjusted for the measures we had of alcohol, physical activity and family history of cardiovascular disease, as well as education and BMI, which influence diet and physical activity, or are influenced by it, and thus may have captured key confounding paths. Of note, our adjustment for confounders is more extensive than previous studies of the association of AMH with lipids and other cardiovascular risk factors. Whilst this is one of the larger studies to explore these associations, our results were imprecisely estimated, with wide confidence intervals, and we are not aware of any independent study that has measures of ovarian reserve and multiple metabolites (or even the amino acids and fatty acids that we observed associations with) in which to attempt to replicate our findings.

Women were awaiting IVF and related to that were confirmed (through cotinine breath test) non-smokers and were of a relatively restricted BMI range. The women were also largely White European and educated to degree level. This homogeneous relatively healthy population may have resulted in some selection bias and may mean that our results do not generalise to a general population of women of reproductive age or other infertile populations. AFC was measured by several operators, and the known intra- and inter-observer variability of AFC [45] and application of a threshold for counting per ovary, with the highest value for AFC of 40 (range 0–40), as compared to AMH of 171 (range 1–171 pmol/l), may explain why we did not see the same strength of associations of AFC with the various metabolites compared to what we observed with AMH. AMH was measured in a single laboratory on an automated analyser with a wide analytical range and low coefficient of variation.

Analyses were undertaken on non-fasting samples. This was necessary to align with clinical processes for a population who are undergoing assisted conception, where caloric restraint may be detrimental. In collaborations of several studies using this same NMR analysis platform results have not differed notably between studies in which the analyses were undertaken in participants who had been advised to fast and those who had not, including in analyses exploring the associations of these metabolites with cardiovascular diseases [14].

The NMR platform used in these analyses covers considerably more of the lipidome than conventional clinical chemistry measures (total cholesterol, LDL-C, HDL-C and triglycerides) that have previously been explored in relation to ovarian reserve, and in addition includes fatty acids, amino acids, glycolysis metabolites, ketone bodies and an inflammatory marker. We acknowledge it misses a high proportion of the currently quantifiable metabolites in human serum/plasma, including markers of energy balance, microbiota metabolism, vitamins, co-factors and xenobiotics, that may be influenced by ovarian reserve. High-throughput analyses of a wider range of metabolites measured by mass spectrometry are possible in epidemiological studies but considerably more expensive than the NMR platform used here, resulting in their use being frequently restricted to subsamples of cohorts [46].

## Conclusion

This study provides novel insight into the association of the ovarian biomarkers, AMH and AFC, with metabolic profiles. Taken together with other recent studies, our results suggest that dyslipidaemia may have a limited role to play in the relationship between ovarian reserve and cardiovascular diseases. The novel associations we find with some fatty acids and amino acids may have a role in mediating any effect of ovarian reserve on cardiometabolic diseases, but these require replication in large prospective studies and we cannot assume that the small number of associations identified are causal.

## Supplementary information

**Additional file 1: TableS1.** List of NMR metabolomic measures assessed in this study, with their units of quantification. **Table S2.** Associations of possible confounders with AMH. **Table S3.** Associations of possible confounders with AFC. **Fig. S1.** Summary of NMR platform used in this paper. **Fig. S2.** Associations of lipoprotein classes with AMH/AFC (unadjusted). **Fig. S3.** Associations of lipoprotein classes and fatty acids with AMH/AFC (unadjusted). **Fig. S4.** Associations of metabolic traits with AMH/AFC (unadjusted). **Fig. S5.** Association of lipoprotein classes with AMH in women with male factor infertility or no male partner. **Fig. S6.** Association of lipoprotein classes and fatty acids with AMH in women with male factor infertility or no male partner. **Fig. S7**. Association of metabolic traits with AMH in women with male factor infertility or no male partner. **Fig. S8.** Association of lipoprotein classes with AFC in women with male factor infertility or no male partner. **Fig. S9.** Association of lipoprotein classes and fatty acids with AFC in women with male factor infertility or no male partner. **Fig. S10** Association of metabolic traits with AFC in women with male factor infertility or no male partner. **Fig. S11.** Comparison of associations for AMH and AFC for whole cohort and with PCOS cases (*n* = 24) removed.

**Additional file 2: Table S4.** Adjusted associations of metabolites with AMH in original units. **Table S5.** Adjusted associations of metabolites with AFC in original units.

## Data Availability

The datasets used and/or analysed during the current study are available from the corresponding author on reasonable request.

## Abbreviations

AFC: Antral follicle count
AMH: Anti-Müllerian hormone
BCAA: Branched-chain amino acid
BMI: Body mass index
DAG: Directed acyclic graph
HDL-C: High-density lipoprotein cholesterol
HOMA-IR: Homeostatic Model Assessment of Insulin Resistance
IQR: Inter-quartile range
IVF: In vitro fertilisation
LDL: Low-density lipoprotein
LDL-C: Low-density lipoprotein cholesterol
NMR: Nuclear magnetic resonance
PCOS: Polycystic ovarian syndrome
PUFA: Polyunsaturated fatty acid
VLDL: Very low-density lipoprotein
