# Supplementary material for: Association of the functional ovarian reserve with serum metabolomic profiling by nuclear magnetic resonance spectroscopy: a cross-sectional study of ~ 400 women
Source: BMC Med. 2020 Aug 31;18:247. doi: 10.1186/s12916-020-01700-z (PMC7457540; doi:10.1186/s12916-020-01700-z)
Supplement: Supplementary file 1 — Additional file 1: TableS1. List of NMR metabolomic measures assessed in this study, with their units of quantification. Table S2. Associations of possible confounders with AMH. Table S3. Associations of possible confounders with AFC. Fig. S1. Summary of NMR platform used in this paper. Fig. S2. Associations of lipoprotein classes with AMH/AFC (unadjusted). Fig. S3. Associations of lipoprotein classes and fatty acids with AMH/AFC (unadjusted). Fig. S4. Associations of metabolic traits with AMH/AFC (unadjusted). Fig. S5. Association of lipoprotein classes with AMH in women with male factor infertility or no male partner. Fig. S6. Association of lipoprotein classes and fatty acids with AMH in women with male factor infertility or no male partner. Fig. S7. Association of metabolic traits with AMH in women with male factor infertility or no male partner. Fig. S8. Association of lipoprotein classes with AFC in women with male factor infertility or no male partner. Fig. S9. Association of lipoprotein classes and fatty acids with AFC in women with male factor infertility or no male partner. Fig. S10 Association of metabolic traits with AFC in women with male factor infertility or no male partner. Fig. S11. Comparison of associations for AMH and AFC for whole cohort and with PCOS cases (n = 24) removed. [file 12916_2020_1700_MOESM1_ESM.docx]

**SUPPLEMENTARY MATERIAL**

**Association of the functional ovarian reserve with serum metabolomic profiling by nuclear magnetic resonance spectroscopy: A cross sectional study of ~400 women.**

Karema Al Rashid MBChB^1†^, Amy Taylor PhD ^2,3,4†^, Mary Ann Lumsden MD^1^, Neil Goulding PhD^2,3^ Deborah A Lawlor PhD^2,3,4^*, Scott M Nelson PhD^1,4^*

^1^ School of Medicine, University of Glasgow, UK G31 2ER

^2^ MRC Integrative Epidemiology Unit at the University of Bristol, UK BS8 2BN
^3^ Population Health Science, Bristol Medical School, UK
^4^ NIHR Bristol Biomedical Research Centre, Bristol, UK

^†^ Joint first authors

* Joint senior authors

| **Content** | **Page(s)** | **Description** |
| --- | --- | --- |
| **Box S1** |  |  |
| NMR platform | 3 | Description of methods for the NMR platform used to quantify metabolic profiles |
| **Tables** |  |  |
| Table S1 | 5 | List of NMR metabolomic measures assessed in this study, with their units of quantification |
| Table S2 | 9 | Associations of possible confounders with AMH |
| Table S3 | 10 | Associations of possible confounders with AFC |
| Table S4 | 11 | Adjusted associations of metabolites with AMH in original units. |
| Table S5 | 11 | Adjusted associations of metabolites with AFC in original units |
| **Figures** |  |  |
| Figure S1 | 12 | Summary of NMR platform used in this paper |
| Figures S2-S4 | 13-15 | Associations of metabolite levels with AMH/AFC (unadjusted) |
| Figures S5-S7 | 16-19 | Association of metabolite levels with AMH in women with male factor infertility or no male partner |
| Figures S8-S10 | 20-23 | Association of metabolite levels with AFC in women with male factor infertility or no male partner |
| Figure S11 | 24 | Comparison of associations for AMH and AFC for whole cohort and with PCOS cases (n=24) removed. |

**Box S1**

**Nuclear Magnetic Resonance (NMR) Spectroscopy Methods**

The NMR spectroscopy methodology is summarised in **Figure S1** below and the metabolites that were quantified, together with the units in which they are quantified, are shown **Table S2**. This approach uses three molecular windows, (two that were applied to native serum and one to serum lipid extracts requiring minimal preparation) to quantify the 158 metabolic traits. The NMR-based metabolite quantification is achieved through measurements of three molecular windows from each serum sample. Two of the spectra (LIPO and LMWM windows) are acquired from native serum and one spectrum from serum lipid extracts (LIPID window). The NMR spectra are measured using Bruker AVANCE III spectrometer operating at 500 or 600 MHz. Measurements of native serum samples and serum lipid extracts are conducted at 37^o^C and 22^O^C, respectively.

The LIPO window represents a standard spectrum of human serum displaying broad overlapping resonances arising from lipid molecules in various lipoprotein particles. The LIPO data are recorded using 8 transients acquired using a NOESY-presat pulse sequence with mixing time of 10ms and water peak suppression. The LMWM window includes signals from various low-molecular-weight molecules. The LMWM spectrum is recorded using a relaxation-filtered pulse sequence that suppresses most of the broad macromolecule and lipid signals to enhance detection of small solutes. Specifically, a Carr-Purcell-Meiboom-Gill (CPMG) pulse sequence with a 78ms *T_2_*-filter and fixed echo delay of 403µs is applied using 24 transients. The LIPID window of the serum extracts is acquired with a standard 1D spectrum using 32 transients.

***QC and outputs***

The NMR spectra were analysed for absolute metabolite quantification (molar concentration) in an automated fashion. For each metabolite a ridge regression model was applied for quantification in order to overcome the problems of heavily overlapping spectral data. In the case of the lipoprotein lipid data, quantification models were calibrated using high performance liquid chromatography methods, and individually cross-validated against NMR-independent lipid data. Low-molecular-weight metabolites, as well as lipid extract measures, were quantified as mmol/l based on regression modelling calibrated against a set of manually fitted metabolite measures. The calibration data are quantified based on iterative line-shape fitting analysis using PERCH NMR software (PERCH Solutions Ltd., Kuopio, Finland). Absolute quantification cannot be directly established for the lipid extract measures due to experimental variation in the lipid extraction protocol. Therefore, serum extract metabolites are scaled via the total cholesterol as quantified from the native serum LIPO spectrum.

**Table S1: NMR metabolic measures**

| **Molecular class** | **Lipid, lipoprotein or metabolite name** | **Units*** |
| --- | --- | --- |
|  |  |  |
| **Extremely large VLDL** | Concentration of chylomicrons and extremely large VLDL particles | mol/l |
|  | Total lipids in chylomicrons and extremely large VLDL | mmol/l |
|  | Phospholipids in chylomicrons and extremely large VLDL | mmol/l |
|  | Total cholesterol in chylomicrons and extremely large VLDL | mmol/l |
|  | Free cholesterol in chylomicrons and extremely large VLDL | mmol/l |
|  | Triglycerides in chylomicrons and extremely large VLDL | mmol/l |
| **Very large VLDL** | Concentration of very large VLDL particles | mol/l |
|  | Total lipids in very large VLDL | mmol/l |
|  | Phospholipids in very large VLDL | mmol/l |
|  | Total cholesterol in very large VLDL | mmol/l |
|  | Cholesterol esters in very large VLDL | mmol/l |
|  | Free cholesterol in very large VLDL | mmol/l |
|  | Triglycerides in very large VLDL | mmol/l |
| **Large VLDL** | Concentration of large VLDL particles | mol/l |
|  | Total lipids in large VLDL | mmol/l |
|  | Phospholipids in large VLDL | mmol/l |
|  | Total cholesterol in large VLDL | mmol/l l |
|  | Cholesterol esters in large VLDL | mmol/l |
|  | Free cholesterol in large VLDL | mmol/l |
|  | Triglycerides in large VLDL | mmol/l |
| **Medium VLDL** | Concentration of large VLDL particles | mol/l |
|  | Total lipids in small VLDL | mmol/l |
|  | Phospholipids in small VLDL | mmol/l |
|  | Total cholesterol in small VLDL | mmol/l |
|  | Cholesterol esters in small VLDL | mmol/l |
|  | Free cholesterol in small VLDL | mmol/l |
|  | Triglycerides in small VLDL | mmol/l |
| **Small VLDL** | Concentration of very small VLDL particles | mol/l |
|  | Total lipids in very small VLDL | mmol/l |
|  | Phospholipids in very small VLDL | mmol/l |
|  | Total cholesterol in very small VLDL | mmol/l |
|  | Cholesterol esters in very small VLDL | mmol/l |
|  | Free cholesterol in very small VLDL | mmol/l |
|  | Triglycerides in very small VLDL | mmol/l |
| **IDI** | Concentration of IDL particles | mol/l |
|  | Total lipids in IDL | mmol/l |
|  | Phospholipids in IDL | mmol/l |
|  | Total cholesterol in IDL | mmol/l |
|  | Cholesterol esters in IDL | mmol/l |
|  | Free cholesterol in IDL | mmol/l |
|  | Triglycerides in IDL | mmol/l |
| **Large LDL** | Concentration of large LDL particles | mol/l |
|  | Total lipids in large LDL | mmol/l |
|  | Phospholipids in large LDL | mmol/l |
|  | Total cholesterol in large LDL | mmol/l |
|  | Cholesterol esters in large LDL | mmol/l |
|  | Free cholesterol in large LDL | mmol/l |
|  | Triglycerides in large LDL | mmol/l |
| **Medium LDL** | Concentration of medium LDL particles | mol/l |
|  | Total lipids in medium LDL | mmol/l |
|  | Phospholipids in medium LDL | mmol/l |
|  | Total cholesterol in medium LDL | mmol/l |
|  | Cholesterol esters in medium LDL | mmol/l |
|  | Free cholesterol in medium LDL | mmol/l |
|  | Triglycerides in medium LDL | mmol/l |
| **Small LDL** | Concentration of small LDL particles | mol/l |
|  | Total lipids in small LDL | mmol/l |
|  | Phospholipids in small LDL | mmol/l |
|  | Total cholesterol in small LDL | mmol/l |
|  | Cholesterol esters in small LDL | mmol/l |
|  | Free cholesterol in small LDL | mmol/l |
|  | Triglycerides in small LDL | mmol/l |
| **Very large HDL** | Concentration of very large HDL particles | mol/l |
|  | Total lipids in very large HDL | mmol/l |
|  | Phospholipids in very large HDL | mmol/l |
|  | Total cholesterol in very large HDL | mmol/l |
|  | Cholesterol esters in very large HDL | mmol/l |
|  | Free cholesterol in very large HDL | mmol/l |
|  | Triglycerides in very large HDL | mmol/l |
| **Large HDL** | Concentration of large HDL particles | mol/l |
|  | Total lipids in large HDL | mmol/l |
|  | Phospholipids in large HDL | mmol/l |
|  | Total cholesterol in large HDL | mmol/l |
|  | Cholesterol esters in large HDL | mmol/l |
|  | Free cholesterol in large HDL | mmol/l |
|  | Triglycerides in large HDL | mmol/l |
| **Medium HDL** | Concentration of medium HDL particles | mol/l |
|  | Total lipids in medium HDL | mmol/l |
|  | Phospholipids in medium HDL | mmol/l |
|  | Total cholesterol in medium HDL | mmol/l |
|  | Cholesterol esters in medium HDL | mmol/l |
|  | Free cholesterol in medium HDL | mmol/l |
|  | Triglycerides in medium HDL | mmol/l |
| **Small HDL** | Concentration of small HDL particles | mol/l |
|  | Total lipids in small HDL | mmol/l |
|  | Phospholipids in small HDL | mmol/l |
|  | Total cholesterol in small HDL | mmol/l |
|  | Cholesterol esters in small HDL | mmol/l |
|  | Free cholesterol in small HDL | mmol/l |
|  | Triglycerides in small HDL | mmol/l |
| **Lipoprotein particle size** | Mean diameter for VLDL particles | nm |
|  | Mean diameter for LDL particles | nm |
|  | Mean diameter for HDL particles | nm |
| **Cholesterol concentrations** | Total cholesterol | mmol/l |
|  | Total cholesterol in VLDL | mmol/l |
|  | Remnant cholesterol (non-HDL and non-LDL cholesterol) | mmol/l |
|  | Total cholesterol in LDL | mmol/l |
|  | Total cholesterol in HDL | mmol/l |
|  | Total cholesterol in HDL2 | mmol/l |
|  | Total cholesterol in HDL3 | mmol/l |
|  | Esterified cholesterol | mmol/l |
|  | Free cholesterol | mmol/l |
| **Glycerides and phospholipid concentrations (and one ratio)** | Total triglycerides | mmol/l |
|  | Triglycerides in VLDL | mmol/l |
|  | Triglycerides in LDL | mmol/l |
|  | Triglycerides in HDL | mmol/l |
|  | Total phosphoglycerides | mmol/l |
|  | Ratio of triglycerides to phosphoglycerides |  |
|  | Phosphatydilcholine and other cholines | mmol/l |
|  | Sphingomyelins | mmol/l |
|  | Total cholines | mmol/l |
| **Apolipoprotein concentrations (and one ratio)** | Apolipoprotein A-1 | g/l |
|  | Apolipoprotein B | g/l |
|  |  |  |
| **Fatty acid concentrations** | Total fatty acids | mmol/l |
|  | Estimated degree of saturation |  |
|  | 22:6, docosahexaenoic acid | mmol/l |
|  | 18:2 linoleic acid | mmol/l |
|  | Omega-3 fatty acids | mmol/l |
|  | Omega-6 fatty acids | mmol/l |
|  | Polyunsaturated fatty acids | mmol/l |
|  | Monounsaturated fatty acids; 16:1, 18:1 | mmol/l |
|  | Saturated fatty acids | mmol/l |
| **Fatty acid ratios** | Ratio of 22:6, docosahexaenoic acid to total fatty acids | % |
|  | Ratio of 18:2 linoleic acid to total fatty acids | % |
|  | Ratio of omega-3 fatty acids to total fatty acids | % |
|  | Ratio of omega-6 fatty acids to total fatty acids | % |
|  | Ratio of polyunsaturated fatty acids to total fatty acids | % |
|  | Ratio of monounsaturated fatty acids to total fatty acids | % |
|  | Ratio of saturated fatty acids to total fatty acids | % |
| **Glycolysis related metabolite** | Glucose | mmol/l |
|  | Lactate | mmol/l |
|  | Pyruvate | mmol/l |
|  | Citrate | mmol/l |
|  | Glycerol | mmol/l |
| **Amino acid concentrations** | Alanine | mmol/l |
|  | Glutamine | mmol/l |
|  | Glycine | mmol/l |
|  | Histidine | mmol/l |
| branched | Isoleucine | mmol/l |
| branched | Leucine | mmol/l |
| branched | Valine | mmol/l |
| aromatic | Phenylalanine | mmol/l |
| aromatic | Tyrosine | mmol/l |
| **Ketone body concentrations** | Acetate | mmol/l |
|  | Acetoacetate | mmol/l |
|  | 3-hydroxybutyrate | mmol/l |
| **Fluid balance marker** | Albumin | mmol/l |
|  | Creatinine | mmol/l |
| **Inflammation marker** | Glycoprotein acetyls, mainly a1-acid glycoprotein | mmol/l |

* These are the units used throughout the paper for each of the metabolic measures, unless we state that we are presenting results in standard deviation (SD) units. Where we present results that are the mean (in control participants) at 16-weeks these are the units. Where we present change in metabolic marker (between 16- to 36-weeks) or difference in change of metabolic markers the units are those listed in the table above per one week of gestational age.

VLDL: very low density lipoprotein; LDL: low density lipoprotein; IDL: intermediate density lipoprotein; HDL: high density lipoprotein

**Supplementary Table S2: Associations of possible confounders with AMH (SD)**

**N=398**

|  | **Difference in mean AMH per unit or category of confounder  (SD) (95% CI)** | **P-value** |
| --- | --- | --- |
| Age (years) | -0.09 (-0.11, -0.07) | <0.001 |
| BMI (kg/m2) | -0.002 (-0.03, 0.03) | 0.91 |
| Education  High School  Undergraduate  Postgraduate | REF  0.21 (-0.01, 0.42)  -0.19 (-0.45, 0.08) | -  0.06  0.17 |
| Physical activity (per category increase) | -0.01 (-0.12, 0.10) | 0.85 |
| Ever smoked | 0.10 (-0.12, 0.33) | 0.32 |
| Alcohol (units per week) | -0.005 (-0.03, 0.01) | 0.60 |
| Family History of cardiometabolic diseases (yes vs no) | -0.17 (-0.37, 0.02) | 0.09 |
| Ethnicity (Non-white vs White European) | 0.33 (-0.02, 0.69) | 0.06 |
| Secondary vs primary infertility | -0.11 (-0.32, 0.11) | 0.33 |
| Duration of infertility (years) | 0.01 (-0.04, 0.06) | 0.59 |
| Endometriosis vs unexplained cause | -0.01 (-0.41,0.39) | 0.96 |
| Tubal vs unexplained cause | 0.24 (-0.10,0.57) | 0.16 |
| Ovulatory vs unexplained cause | 0.93 (0.48, 1.37) | <0.001 |
| Male factors vs unexplained cause | 0.03 (-0.21, 0.28) | 0.78 |

**Supplementary Table S3: Associations of possible confounders with Total AFC (SD)**

**N=398**

|  | **Difference in mean AFC per unit or category of confounder  (SD) (95% CI)** | **P-value** |
| --- | --- | --- |
| Age (years) | -0.06 (-0.08, -0.04) | <0.001 |
| BMI (kg/m2) | -0.01 (-0.04, 0.02) | 0.61 |
| Education  High School  Undergraduate  Postgraduate | REF  -0.07 (-0.29, 0.15)  -0.17 (-0.45, 0.10) | -  0.54  0.21 |
| Physical activity (per category increase) | 0.004 (-0.11, 0.12) | 0.94 |
| Ever smoked | -0.14 (-0.36, 0.08) | 0.22 |
| Alcohol (units per week) | -0.02 (-0.04, 0.002) | 0.07 |
| Family History of cardiometabolic diseases (yes vs no) | -0.26 (-0.45, -0.06) | 0.01 |
| Ethnicity (Non-white vs White European) | 0.31 (-0.04, 0.67) | 0.09 |
| Secondary vs primary infertility | -0.34 (-0.55, -0.13) | 0.001 |
| Duration of infertility (years) | -0.005 (-0.06, 0.04) | 0.83 |
| Endometriosis vs unexplained cause | 0.01 (-0.39, 0.41) | 0.96 |
| Tubal vs unexplained cause | 0.14 (-0.20, 0.48) | 0.41 |
| Ovulatory vs unexplained cause | 0.65 (0.20, 1.11) | 0.005 |
| Male factors vs unexplained cause | 0.12 (-0.13, 0.38) | 0.34 |

**Table S4: Adjusted associations of metabolites with AMH in original units**

Adjusted for age, education, family history of CVD, BMI, physical activity, alcohol (units per week), ever smoking, ethnicity, duration of infertility, cause of infertility, primary/secondary infertility.

See linked excel sheet.

**Table S5:** **Adjusted associations of metabolites with AMH in original units**

Adjusted for age, education, family history of CVD, BMI, physical activity, alcohol (units per week), ever smoking, ethnicity, duration of infertility, cause of infertility, primary/secondary infertility.

See linked excel sheet.

**Supplementary Figures**

**Supplemental Figure S1: Stages and methods used for NMR platform metabolic measures (adapted from Wurtz et al. [16])**

LMWM


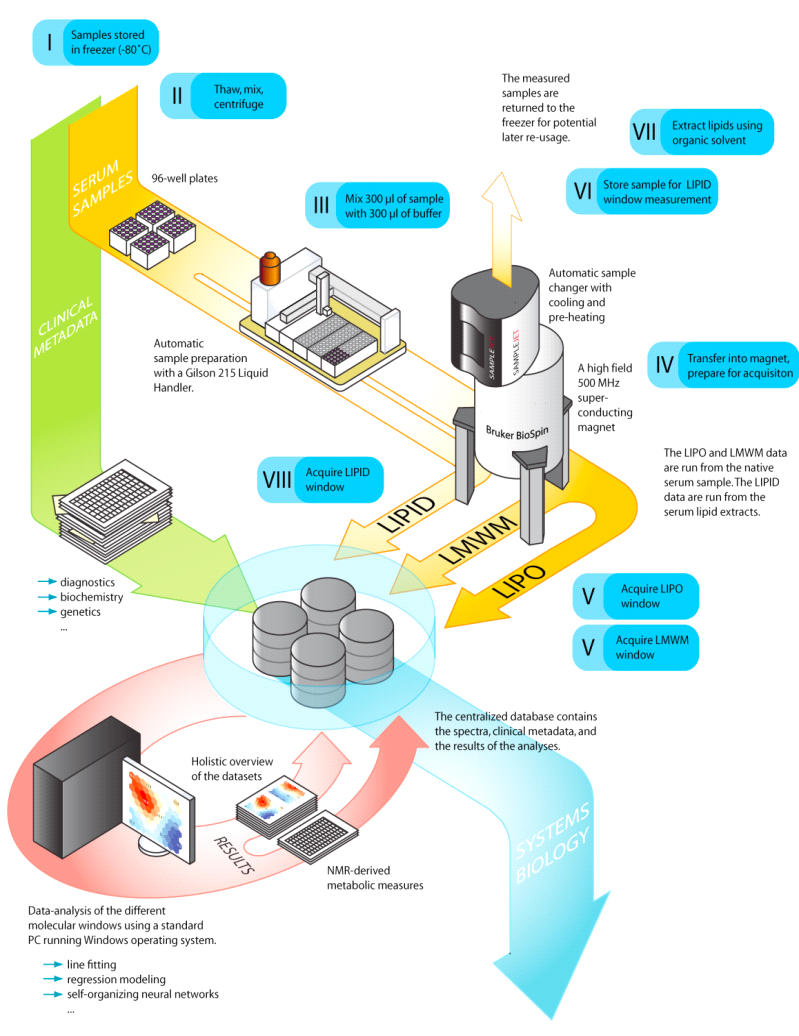

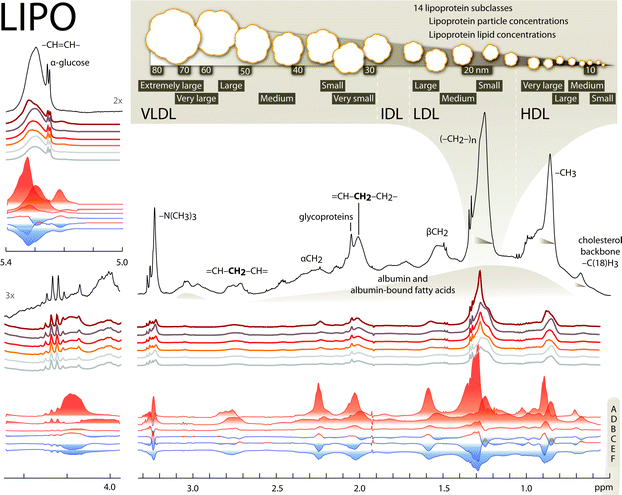

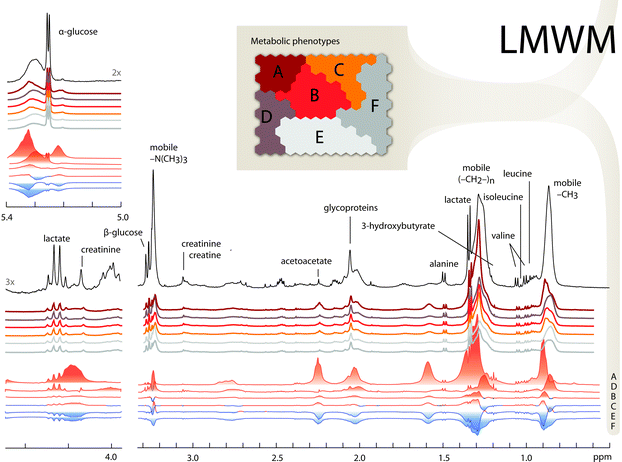


**Supplemental Figure S2-S4. Associations of metabolite levels with AMH/AFC (unadjusted)**

Effect sizes per 1 SD in metabolite concentrations and respective 95% confidence intervals are shown for AMH (red) and AFC (black).

**
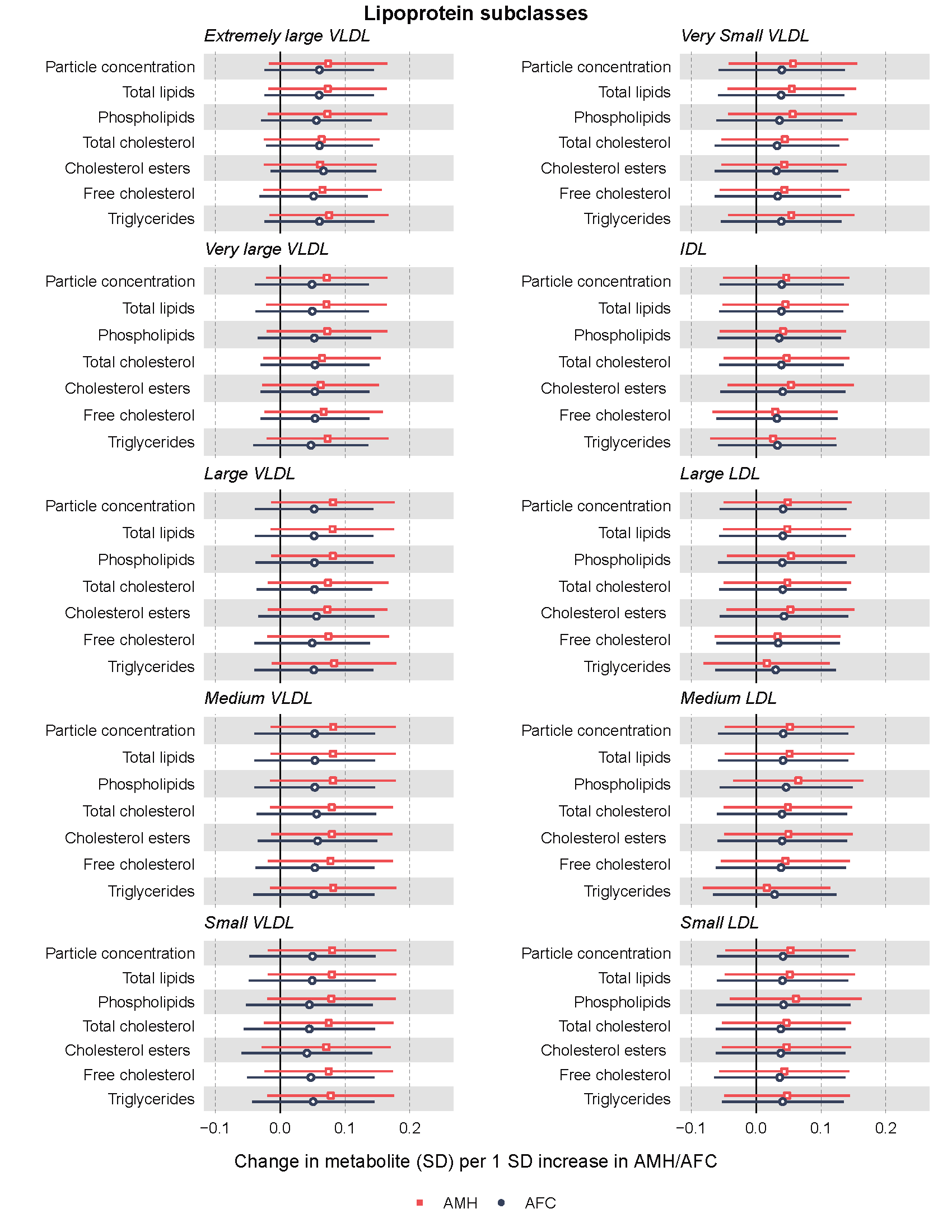
**

**
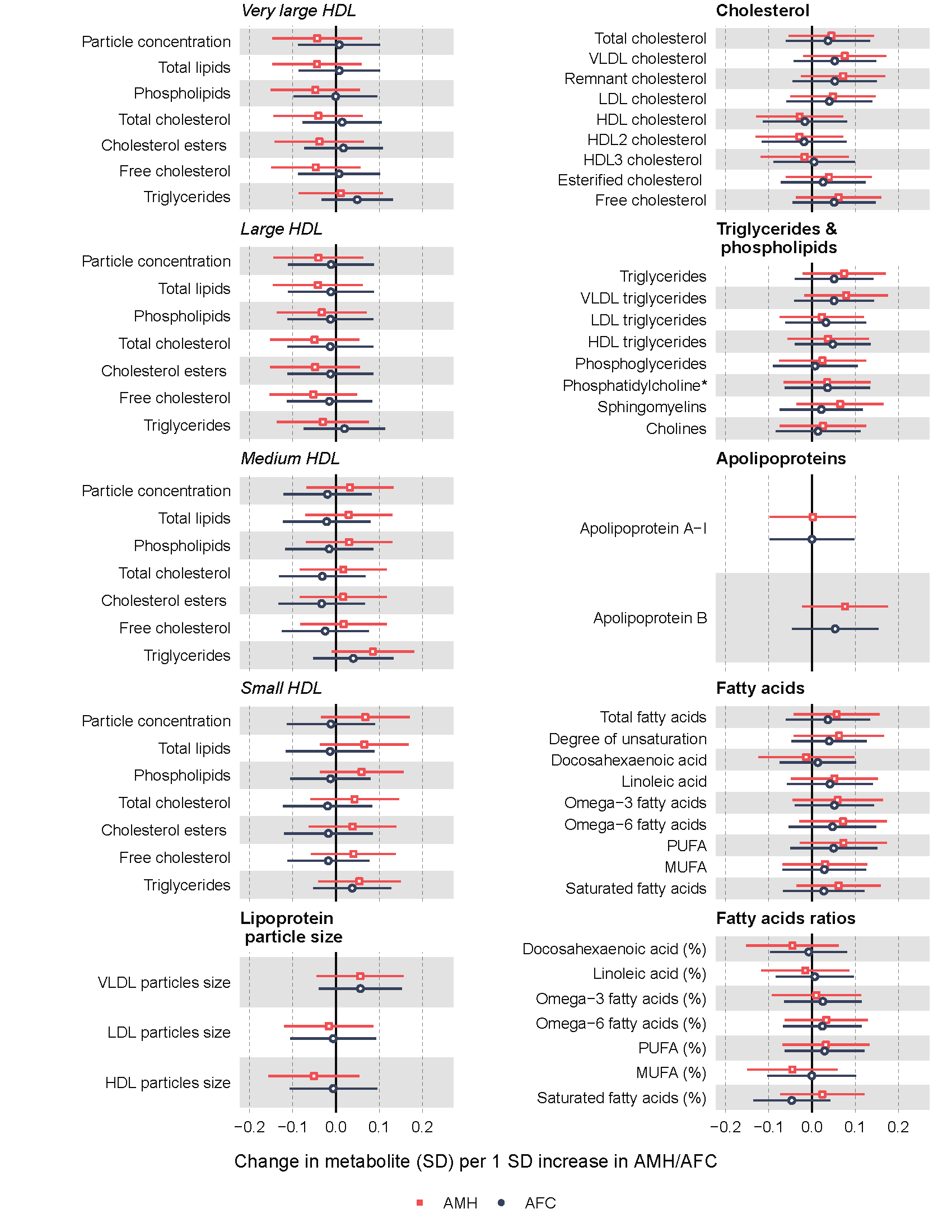

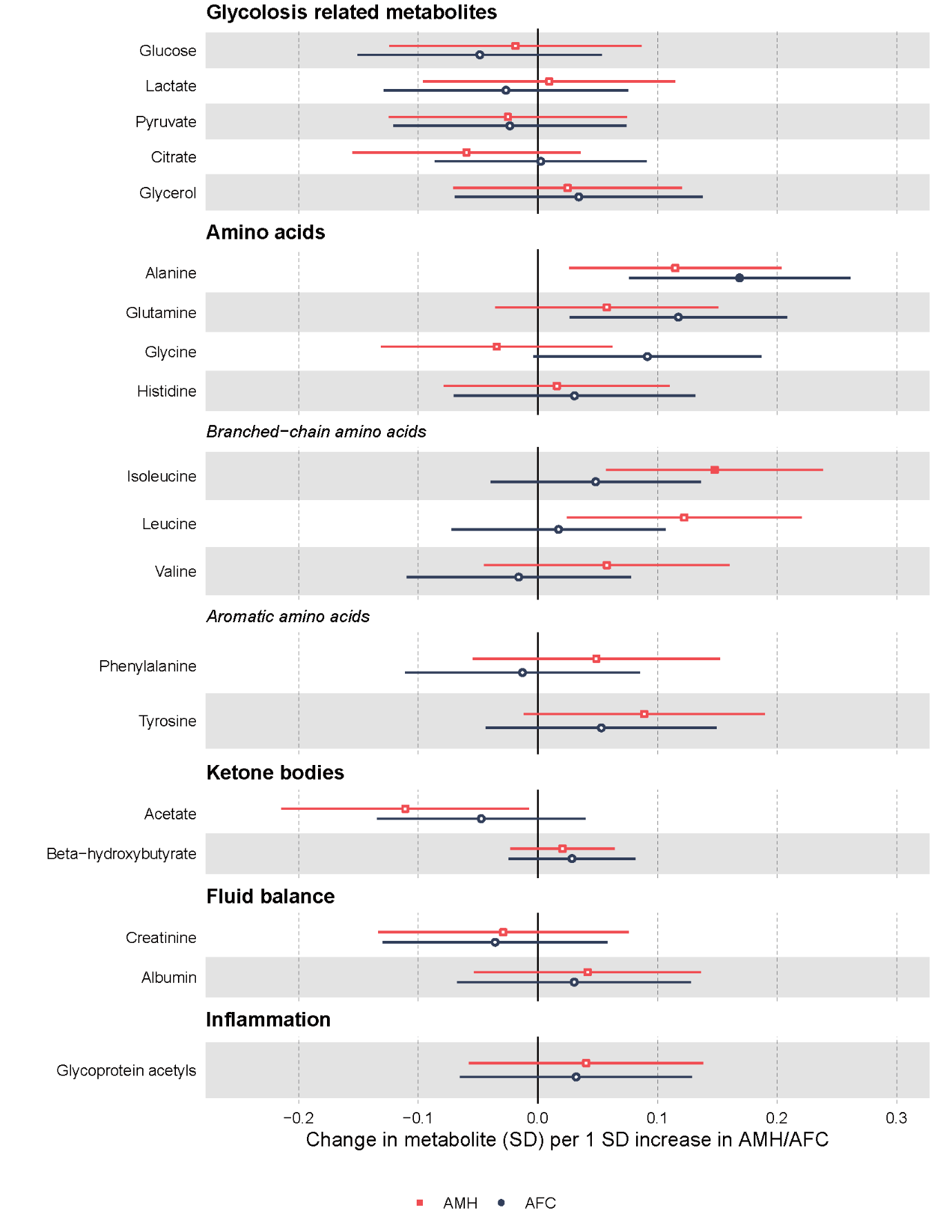
**

**Supplemental Figure S5-S7. Association of metabolite levels with AMH in women with male factor infertility or no male partner**

Effect sizes per 1 SD in metabolite concentrations and respective 95% confidence intervals are shown for AMH (red) and AFC (black). Adjusted for age, education, family history of CVD, BMI, physical activity, alcohol (units per week), ever smoking, ethnicity, duration of infertility, primary/secondary infertility.


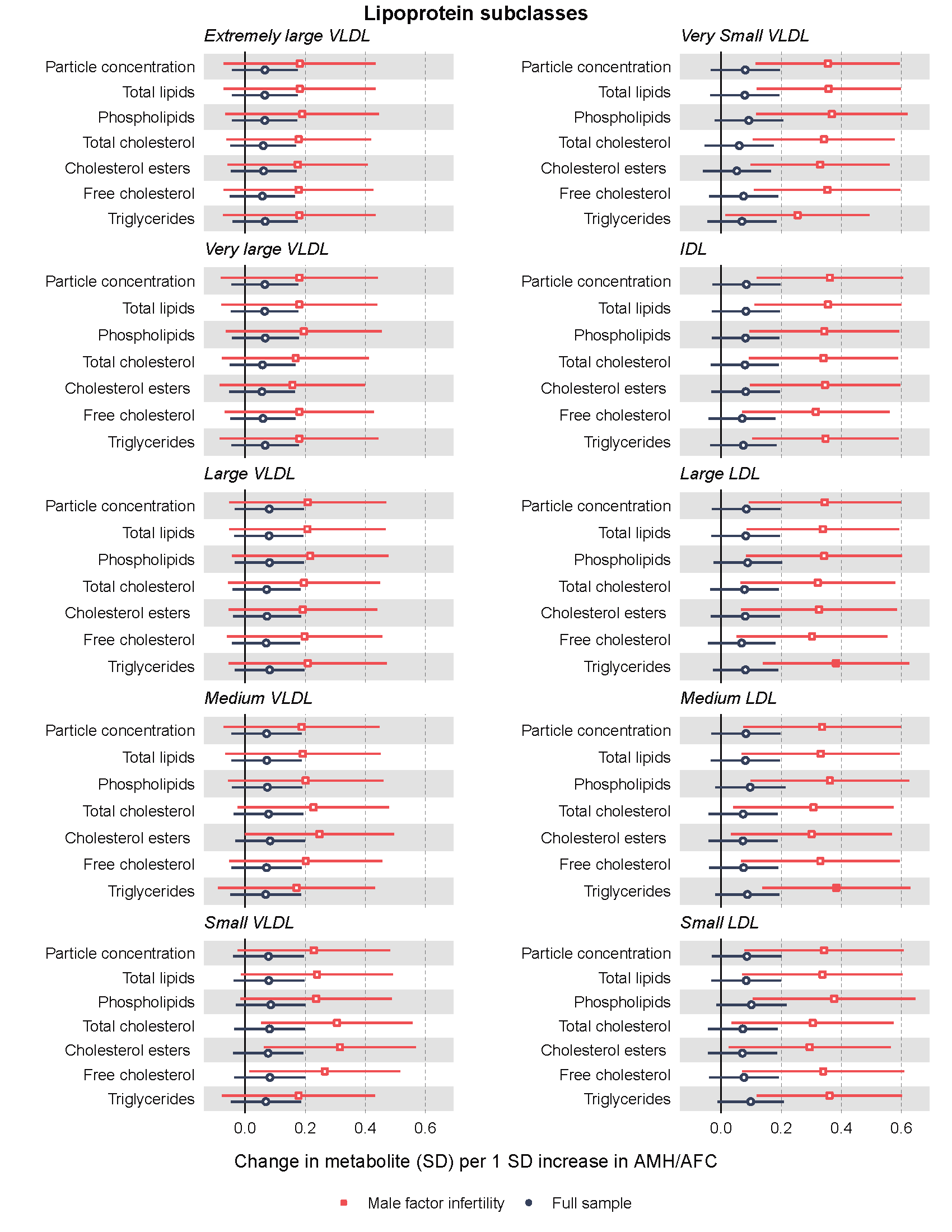


.


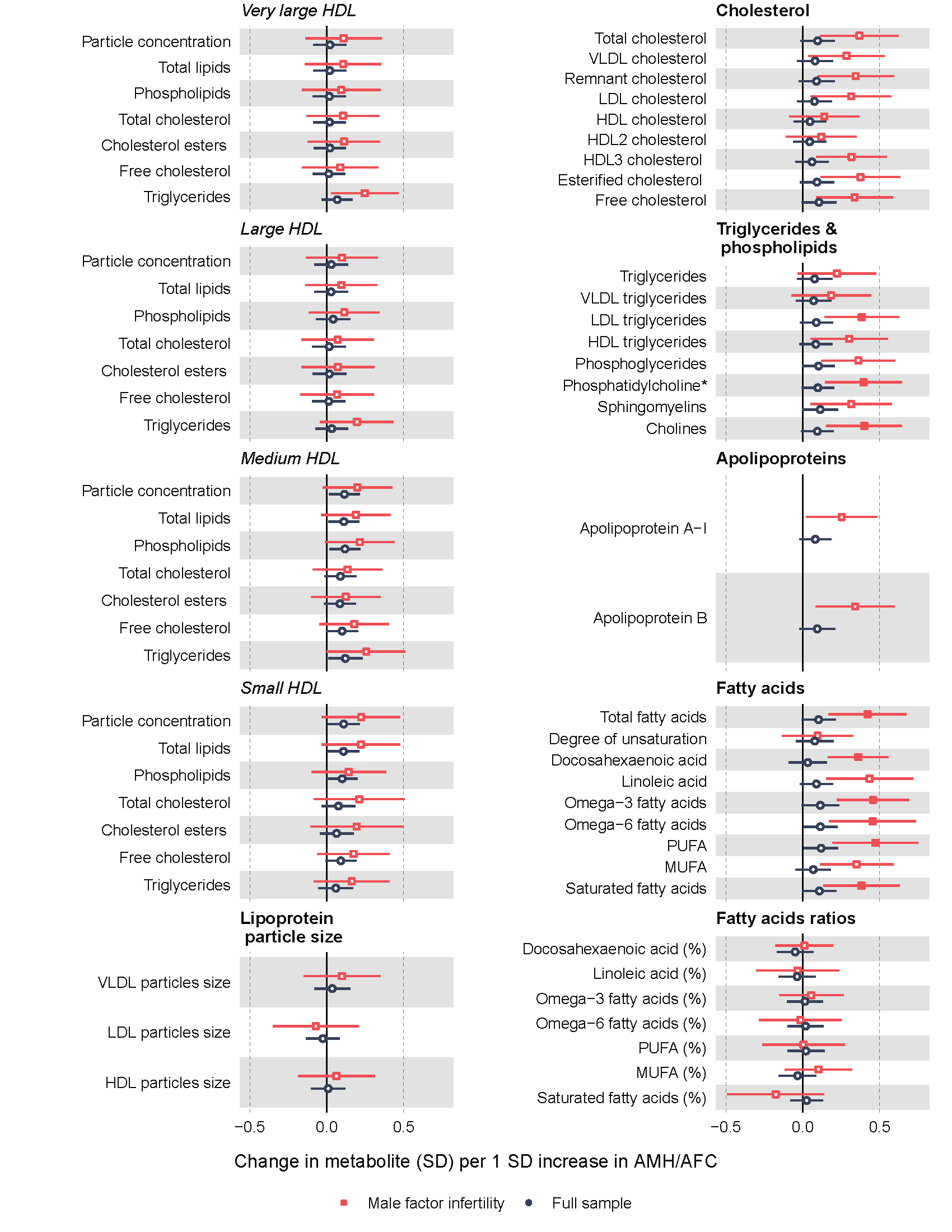

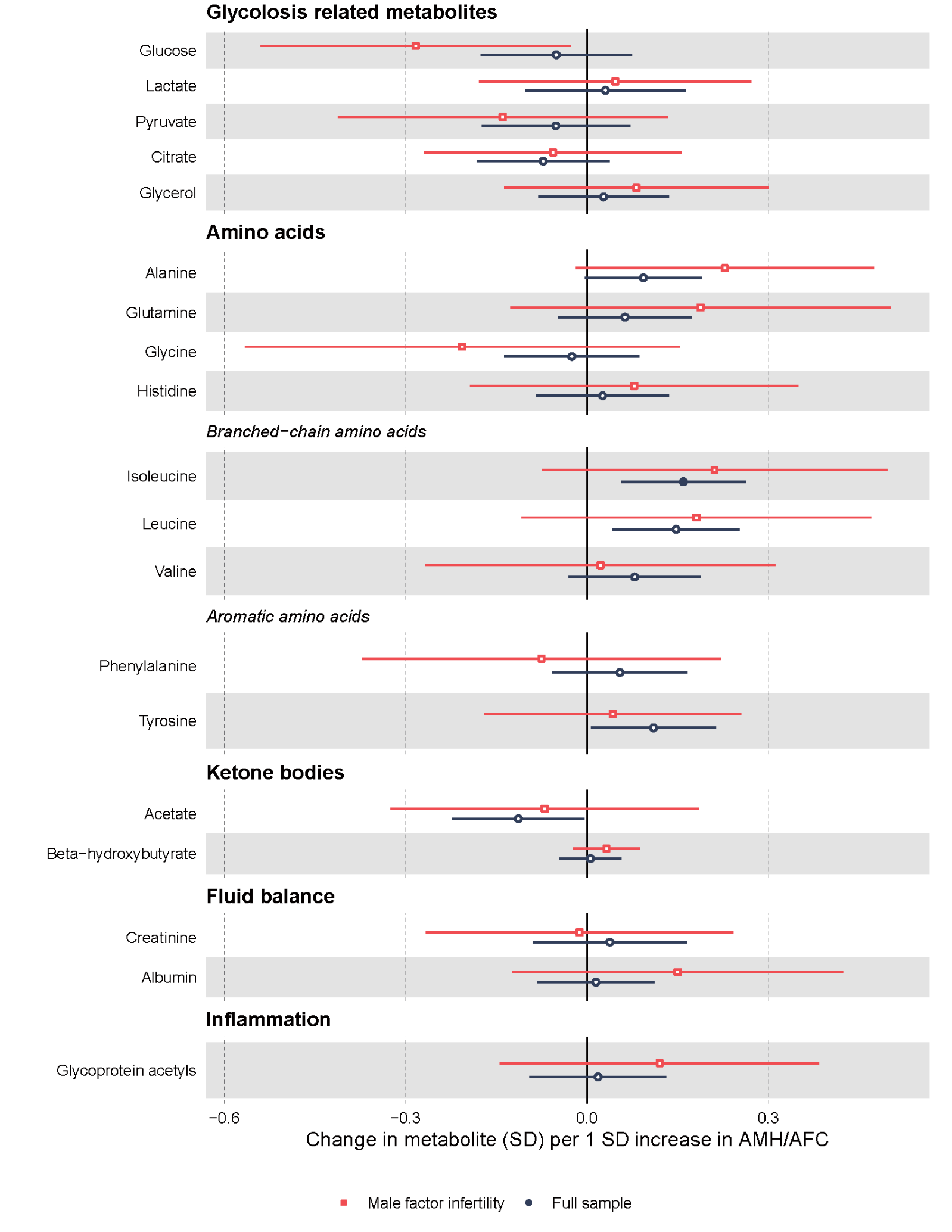


**Supplemental Figure S8-S10. Association of metabolite levels with AFC in women with male factor infertility or no male partner**

Effect sizes per 1 SD in metabolite concentrations and respective 95% confidence intervals are shown for AMH (red) and AFC (black). Adjusted for age, education, family history of CVD, BMI, physical activity, alcohol (units per week), ever smoking, ethnicity, duration of infertility, primary/secondary infertility.


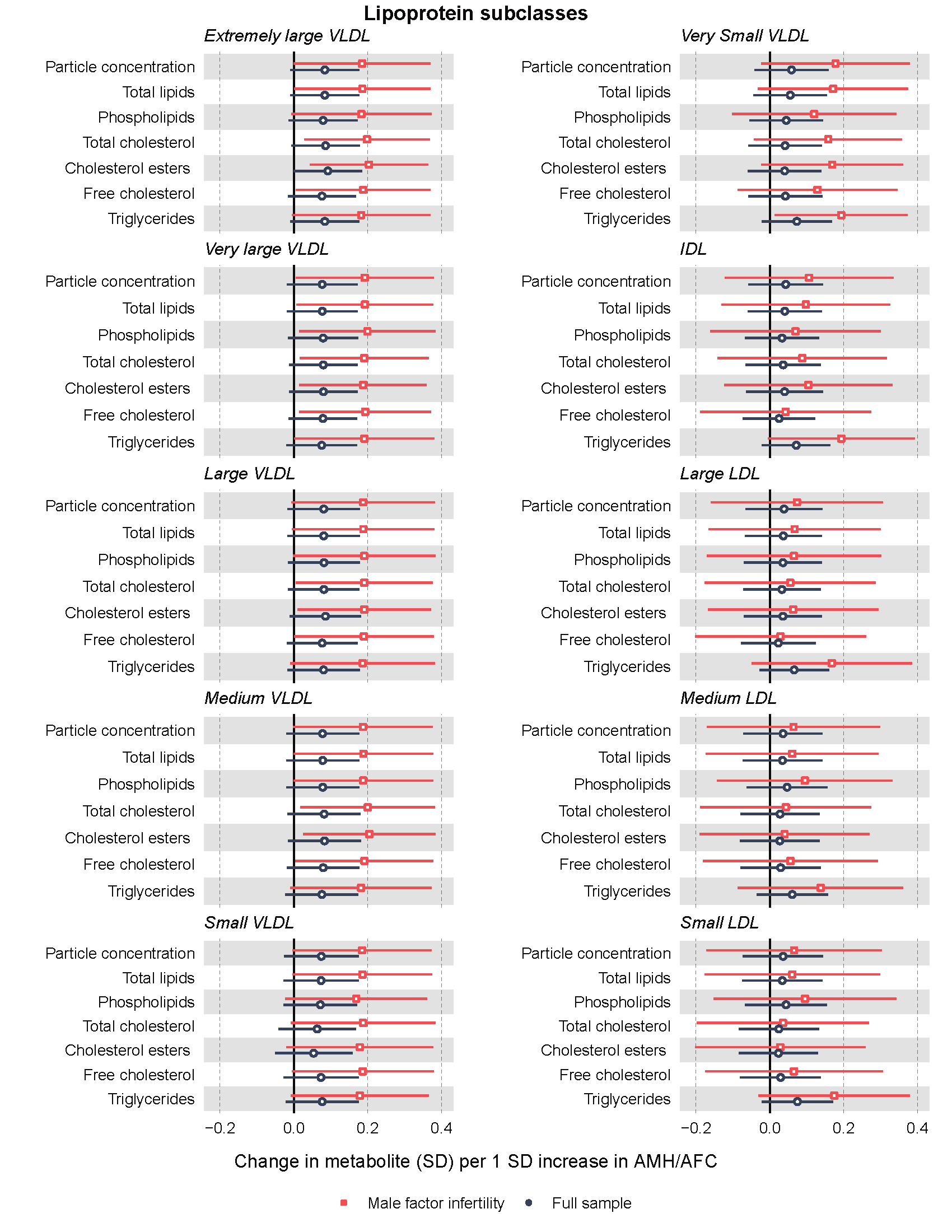


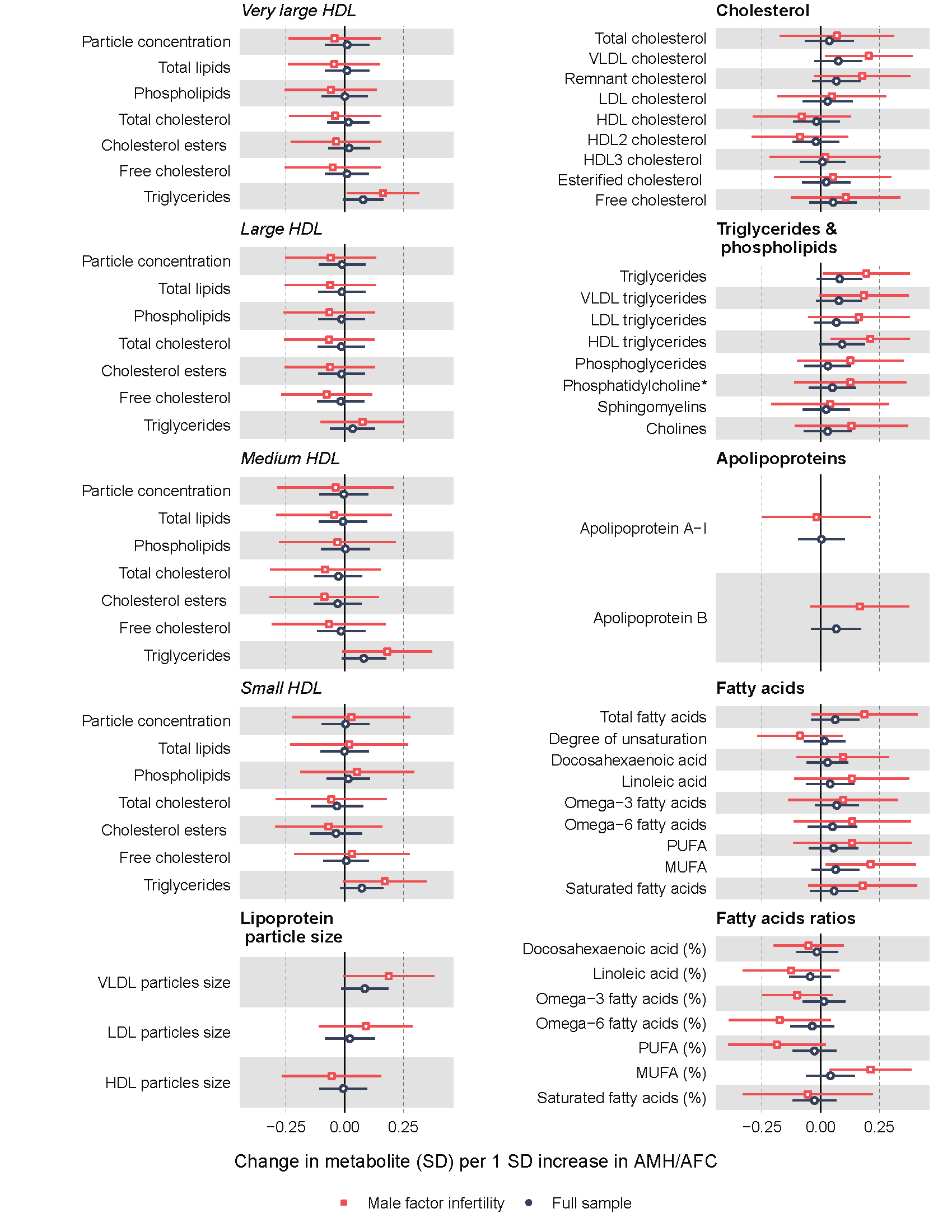

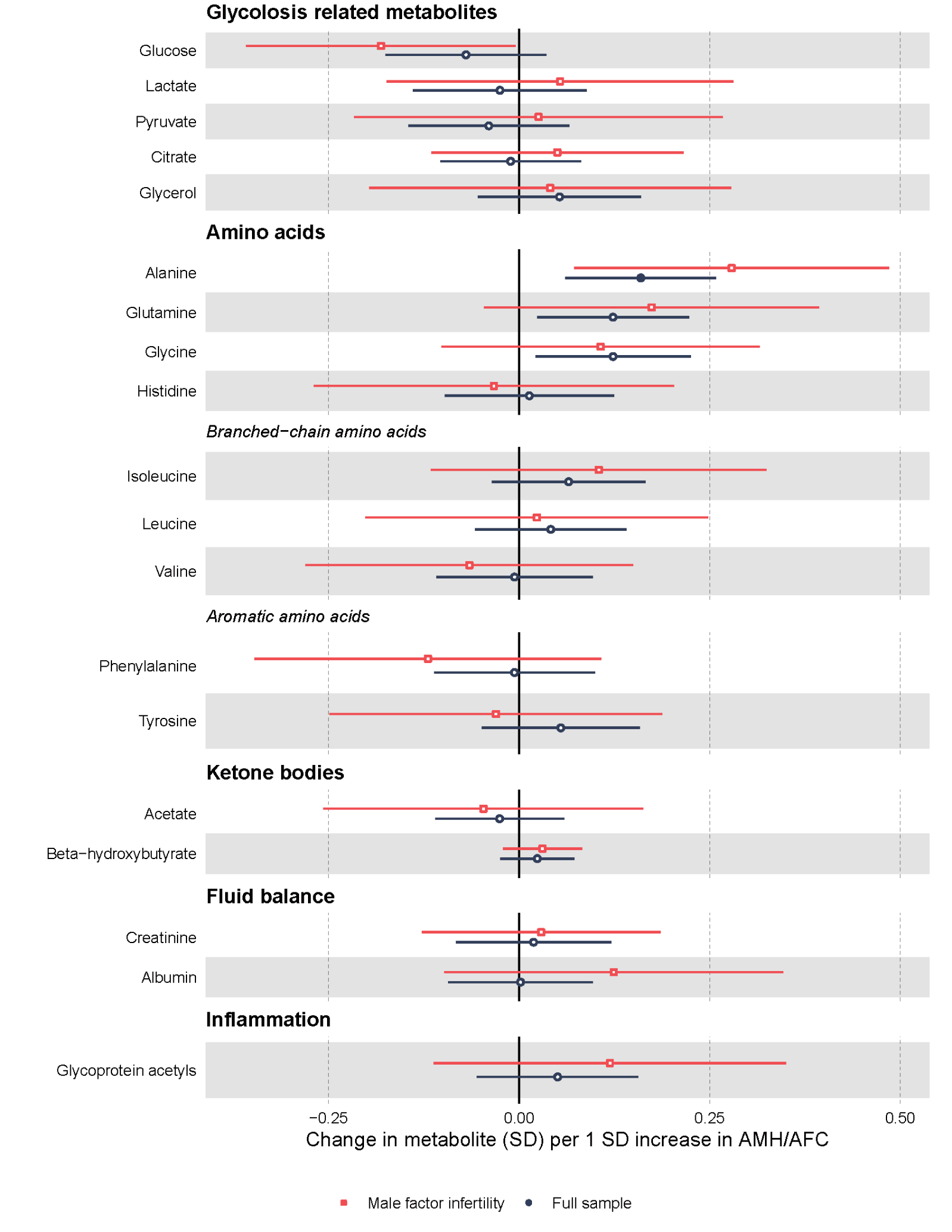


**Supplemental Figure 11: Comparison of associations for AMH and AFC for whole cohort and with PCOS cases (n=24) removed.**
